# The dangers of rubella virus

**DOI:** 10.7554/eLife.89265

**Published:** 2023-06-16

**Authors:** Ekaterina Epifanova, Laurent Nguyen

**Affiliations:** 1 Laboratory of Molecular Regulation of Neurogenesis, GIGA‐Stem Cells and GIGA-Neurosciences, Interdisciplinary Cluster for Applied Genoproteomics (GIGA‐R), University of Liège Liège Belgium; 2 WELBIO department, WEL Research Institute Wavres Belgium

**Keywords:** rubella, organoid, microglia, brain development

## Abstract

The rubella virus can interfere with fetal brain development by infecting immune cells called microglia during pregnancy.

**Related research article** Popova G, Retallack H, Kim CN, Shin D, Wang A, DeRisi J, Nowakowski TJ. 2023. Rubella virus tropism and single cell responses in human primary tissue and microglia-containing organoids. *eLife* **12**:RP87696. doi: 10.7554/eLife.87696.

Some pathogens can be extremely harmfull during pregnancy as they can cross the placenta, infect the fetus, and go on to cause congenital birth defects, miscarriages and stillbirths (Pereira, 2018). The rubella virus, for example, can cause a range of congenital brain defects, and it is also associated with a higher risk of babies developing congenital rubella syndrome, a complex condition associated with developmental delays, cardiac anomalies, hearing impairment and eye abnormalities (Bardeletti et al., 1975; Lazar et al., 2016; Toizumi et al., 2017). These viruses and other pathogens are collectively referred to as TORCH pathogens, which is short for toxoplasmosis (which is caused by a parasite), other pathogens (such as syphilis, varicella, mumps, parvovirus B19 and HIV), rubella, cytomegalovirus, and herpes simplex virus.

Despite the threat they pose to public health, the mechanisms by which TORCH pathogens affect brain development remain poorly understood. Now, in eLife, Tomasz Nowakowski, Joseph DeRisi and colleagues at the University of California San Francisco – including Galina Popova and Hanna Retallack as joint first authors – report new insights into the infection of brain cells by the rubella virus (Popova et al., 2023).

The researchers combined analyses of live human fetal brain slices that were maintained in the laboratory and two-dimensional cell cultures to study how the rubella virus affects brain cells. This revealed that the virus predominantly infects immune cells called microglia, which patrol and scavenge the central nervous system for damaged cells and pathogens. Microglia also have an important role in protecting the brain during development.

The experiments revealed that the rubella virus can only infect microglia when a variety of other brain cells are present. This is likely due to some yet-to-be-identified diffusible factors released by the other brain cells, which could render microglia susceptible to infection. However, the microglia do not need to make direct contact with these other cells in order to get infected.

Microglia play a crucial role in the antiviral immune response by releasing inflammatory cytokines, such as interferons (Sala and Kuka, 2020). Popova et al. found that infection with the rubella virus leads to an excessive interferon response by neighbouring neuronal cells, and this could have a deleterious effect on brain development. This is consistent with previous research, which showed that prenatal infection with rubella and HIV can trigger the overproduction of interferons, leading to prolonged inflammation that may contribute to the atypical development of the fetus (Crow et al., 2003). Certain inflammatory disorders, such as systemic lupus erythematosus and Aicardi-Goutières syndrome, are also characterized by an increased interferon response, and it is possible that some TORCH infections (in particular HIV and Rubella) share certain phenotypic similarities with these conditions.

As with many other TORCH pathogens, the fetus is most vulnerable to the rubella virus during the first trimester of pregnancy, due to the lack of immune defense in the developing fetus. Microglia populate the brain about a month into pregnancy, and the blood-brain barrier in the fetal brain only starts to be functional about two months into pregnancy (Menassa and Gomez-Nicola, 2018; Goasdoué et al., 2017). The brain is therefore extremely vulnerable to viruses during the first trimester of pregnancy, which coincides with a higher risk of developing severe developmental disorders.

The study of Popova et al. highlights the importance of using human cell-based models to better understand the pathophysiological mechanisms of congenital rubella syndrome. It remains to be seen why and how the rubella virus specifically attacks microglia, and what its molecular targets are. Identifying the molecular cues released by other brain cells, which potentially increase infection, will be necessary to eventually develop therapies against congenital rubella syndrome.

**Figure 1.**
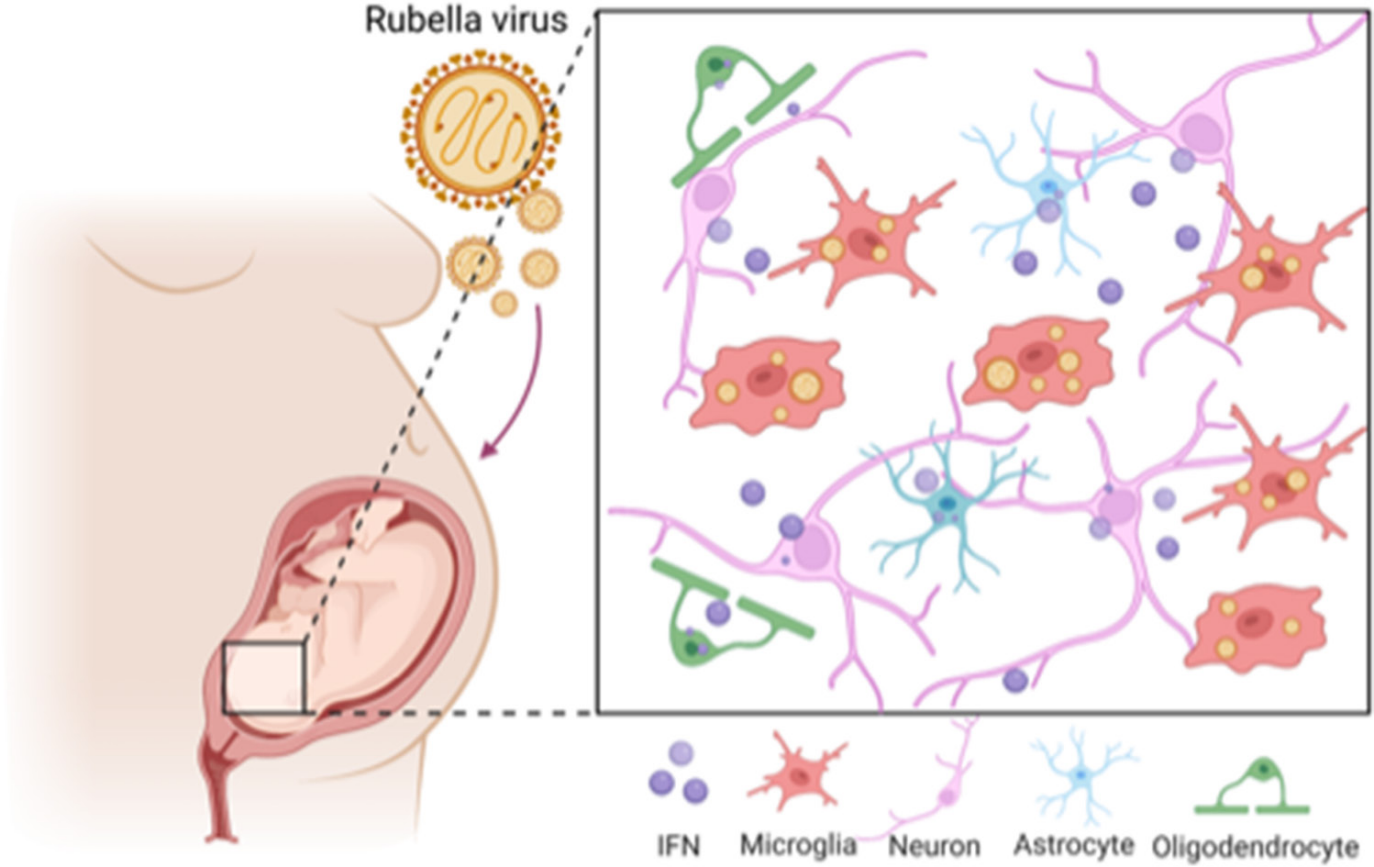
The effect of rubella virus on the brain development. Rubella viruses (orange circles) target immune cells, called microglia (red), in the brain of the fetus. Popova et al. show that other neighbouring brain cells (pink, green and blue) must be present for infection to take place: it is thought that infection relies on diffusible factors released by these cells. Infection causes the release of large amounts of a signaling protein, called interferon (IFN; purple), which can damage the developing brain.
